# Supplementary material for: Oilseed Rape Shares Abundant and Generalized Pollinators with Its Co-Flowering Plant Species
Source: Insects. 2021 Dec 8;12(12):1096. doi: 10.3390/insects12121096 (PMC8704917; doi:10.3390/insects12121096)
Supplement: Supplementary file 1 [file insects-12-01096-s001.zip › insects-1482118-SI.pdf]

SUPPLEMENTARY MATERIALS

Figure S1. Results from Müller's Index calculated using the PAC function in R. Index ranges from 0 to 1 with 0 indicatting no shared visitors (no influence) to 1, diet of all visitors depends on the acting plant. The values for *Brassica napus*' effect on plants in the community (a) and the effect of other plant species on *Brassica napus* (b).

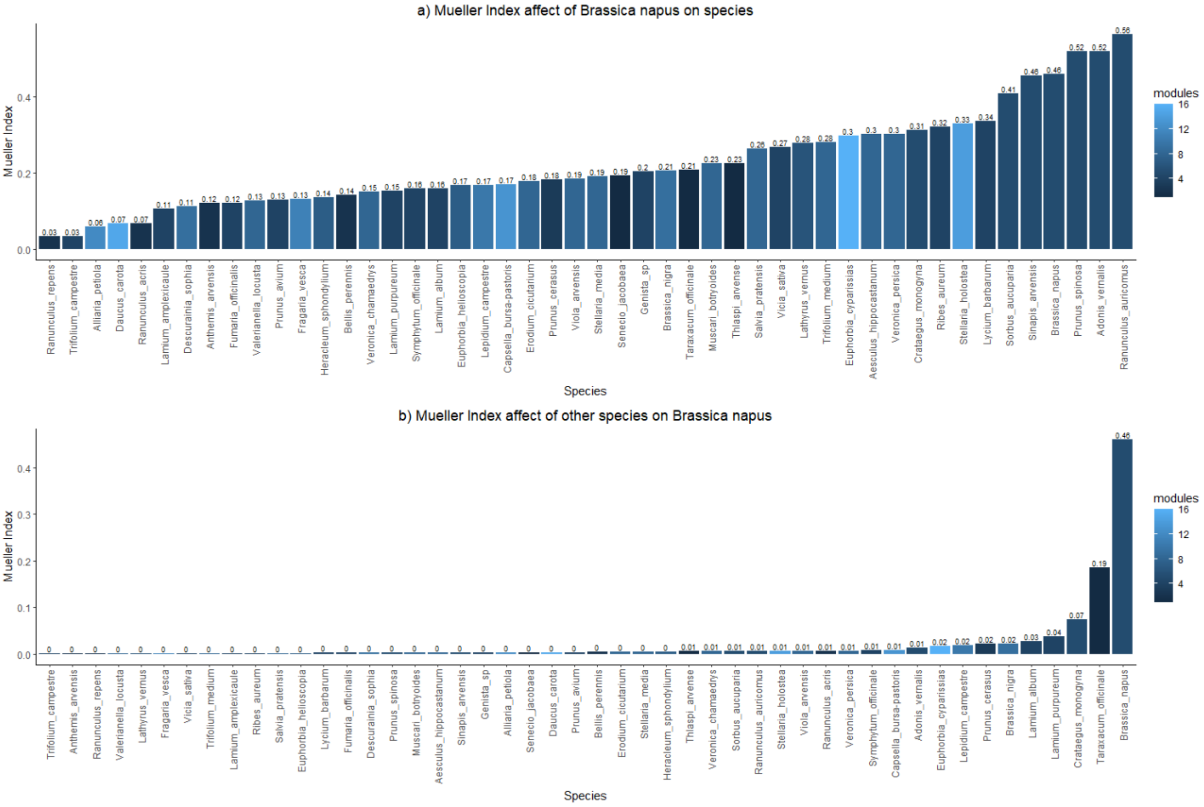

Table S1. Information of sites sampled including coordinates (WGS84), the number of interactions (Int), pollinator species (Pol), plant species (Pl) and time spent sampling (hours:minutes) at each site.

Site	ID	Lat	Long	Int	Pol	Pl	Time Sampled
Friedeberg	FRI1	51.62053	11.72198	108	38	13	4:38
Friedeberg	FRI2	51.62335	11.73013	86	29	10	3:54
Friedeberg	FRI3	51.63262	11.70642	141	48	16	4:30
Greifenhagen	GRE1	51.62443	11.4552	166	43	13	4:45
Greifenhagen	GRE2	51.62147	11.45708	26	12	4	0:40
Greifenhagen	GRE3	51.61945	11.43882	197	36	15	4:35
Greifenhagen	GRE4	51.64473	11.43882	172	44	15	5:12
Harsleben	HAR1	51.85657	11.07052	125	22	9	3:39
Harsleben	HAR2	51.83783	11.05842	41	6	4	0:56
Harsleben	HAR3	51.8536	11.08758	157	40	15	4:23
Harsleben	HAR4	51.8259	11.067	71	14	8	2:13
Siptenfelde	SIP1	51.6543	11.03887	195	29	7	3:43
Siptenfelde	SIP2	51.63853	11.0606	182	39	13	4:01
Siptenfelde	SIP3	51.6365	11.05487	164	22	9	3:50
Schafstädt	SST1	51.38192	11.71798	147	36	13	4:30
Schafstädt	SST2	51.37763	11.72055	94	24	11	2:55
Schafstädt	SST3	51.35478	11.73462	237	25	9	3:30
Wanzleben	WAN1	52.09462	11.4175	143	33	16	4:20
Wanzleben	WAN2	52.09283	11.4278	178	27	9	4:18
Wanzleben	WAN3	52.08375	11.41837	148	31	10	3:44

Table S2. Extrapolated species richness based on number of individuals (for pollinators) and number of interactions sampled using specpool in the vegan package. Observed richness; Estimated richness with Chao1 estimator; Standard error with Chao1 estimator; Sampling completeness (Observed richness/Estimated richness x 100 ± Standard Error) for plant species, pollinator species and interactions of both.

	Observed Richness	Estimated Richness	Standard Error	Sampling Completeness (%)
Pollinator Species	189	281.75	27.02	61.21-74.19
Interactions	567	1836.55	202.58	27.81-34.70

Table S3. Relative abundance of functional groups for a) plants and b) pollinators used in analyses. Relative abundance was caculated as number of interactions observed for a functional group divided by total number of interactions in network; then multiplied by 100 for a percentage.

a) Plant functional groups

**Flower Type**	**Relative abundance**
Bell	1.80 %
Disk with Hidden Nectar (HN)	46.04 %
Disk with Open Nectar (ON)	10.44 %
Flag Blossoms	1.19 %
Flower Head	25.23 %
Funnel	0.14 %
Lip	14.33 %
Pollen	0.68 %
Stalk Disk	0.14 %

b) Pollinator functional groups

**Pollinator group**	**Relative abundance**
Bumblebee	19.44 %
Coleoptera	13.46 %
Honeybee	13.00 %
Hoverfly	5.94 %
Lepidoptera	3.42 %
Other_fly	17.93 %
Wasp	4.28 %
Wild_bee	22.53 %

Table S4. List of 48 plant species (in alphabetical order) found in our study along with information for each on its flower type, the number of interactions with pollinators observed, and its module. Each plant is given a short ID, and these IDs are used in Figure 4, which shows network modularity.

ID	Plant	Flower type	Interactions	(Percentage)	Module
P_01	*Adonis vernalis*	Pollen	19	(0.68)	5
P_02	*Aesculus hippocastanum*	Lip	6	(0.22)	8
P_03	*Alliaria petiola*	Disk_HN	32	(1.15)	12
P_04	*Anthemis arvensis*	FlowerHead	2	(0.07)	2
P_05	*Bellis perennis*	FlowerHead	21	(0.76)	2
P_06	*Brassica napus*	Disk_HN	747	(26.89)	5
P_07	*Brassica nigra*	Disk_HN	82	(2.95)	9
P_08	*Capsella bursa-pastoris*	Disk_HN	37	(1.33)	13
P_09	*Crataegus monogyna*	Disk_ON	179	(6.44)	5
P_10	*Daucus carota*	Disk_ON	35	(1.26)	15
P_11	*Descurainia sophia*	Disk_HN	13	(0.47)	9
P_12	*Erodium cicutarium*	Disk_HN	17	(0.61)	8
P_13	*Euphorbia cyparissias*	Disk_ON	40	(1.44)	16
P_14	*Euphorbia helioscopia*	Disk_ON	7	(0.25)	7
P_15	*Fragaria vesca*	Disk_HN	5	(0.18)	11
P_16	*Fumaria officinalis*	FlagBlossoms	12	(0.43)	4
P_17	*Genista sp*	FlagBlossoms	9	(0.32)	4
P_18	*Heracleum sphondylium*	Disk_ON	25	(0.90)	7
P_19	*Lamium album*	Lip	123	(4.43)	4
P_20	*Lamium amplexicaule*	Lip	8	(0.29)	4
P_21	*Lamium purpureum*	Lip	188	(6.77)	4
P_22	*Lathyrus vernus*	FlagBlossoms	2	(0.07)	6
P_23	*Lepidium campestre*	Disk_HN	79	(2.84)	10
P_24	*Lycium barbarum*	StalkDisk	4	(0.14)	4
P_25	*Muscari botryoides*	Bell	8	(0.29)	8
P_26	*Prunus avium*	Disk_HN	19	(0.68)	4
P_27	*Prunus cerasus*	Disk_HN	92	(3.31)	3
P_28	*Prunus spinosa*	Disk_HN	3	(0.11)	5
P_29	*Ranunculus acris*	Disk_HN	72	(2.59)	2
P_30	*Ranunculus auricomus*	Disk_HN	8	(0.29)	5
P_31	*Ranunculus repens*	Disk_HN	14	(0.50)	2
P_32	*Ribes aureum*	Bell	3	(0.11)	4
P_33	*Salvia pratensis*	Lip	4	(0.14)	8

P_34	*Senecio jacobaea*	FlowerHead	12	(0.43)	1
P_35	*Sinapis arvensis*	Disk_ON	4	(0.14)	5
P_36	*Sorbus aucuparia*	Disk_HN	10	(0.36)	5
P_37	*Stellaria holostea*	Disk_HN	14	(0.50)	14
P_38	*Stellaria media*	Disk_HN	17	(0.61)	8
P_39	*Symphytum officinale*	Bell	39	(1.40)	4
P_40	*Taraxacum officinale*	FlowerHead	666	(23.97)	1
P_41	*Thlaspi arvense*	Disk_HN	18	(0.65)	1
P_42	*Trifolium campestre*	FlagBlossoms	4	(0.14)	4
P_43	*Trifolium medium*	FlagBlossoms	3	(0.11)	8
P_44	*Valerianella locusta*	Funnel	4	(0.14)	8
P_45	*Veronica chamaedrys*	Lip	27	(0.97)	8
P_46	*Veronica persica*	Lip	17	(0.61)	8
P_47	*Vicia sativa*	FlagBlossoms	3	(0.11)	4
P_48	*Viola arvensis*	Lip	25	(0.90)	8

Table S5. List of 189 pollinators (in alphabetical order) observed in our study at the highest level of identification along with information for each on its functional group, the number of interactions with plants observed, and its module. Each pollinator is given a short ID, and these IDs are used in Figure 4, which shows network modularity.

ID	Pollinator	Pollinator Group	Interactions	(Percentage)	Module
I_01	Adelidae	Lepidoptera	34	(1.22)	12
I_02	*Aglais io*	Lepidoptera	11	(0.4)	1
I_03	*Alocerus sp*	Coleoptera	1	(0.04)	7
I_04	*Altica sp*	Coleoptera	2	(0.07)	16
I_05	*Ampedus rufipennis*	Coleoptera	1	(0.04)	15
I_06	*Anatis ocellata*	Coleoptera	6	(0.22)	5
I_07	*Andrena alfkenella*	Wild_bee	1	(0.04)	5
I_08	*Andrena chrysosceles*	Wild_bee	4	(0.14)	1
I_09	*Andrena cineraria*	Wild_bee	165	(5.94)	1
I_10	*Andrena dorsata*	Wild_bee	2	(0.07)	5
I_11	*Andrena flavipes*	Wild_bee	74	(2.66)	1
I_12	*Andrena floricola*	Wild_bee	5	(0.18)	10
I_13	*Andrena fucata*	Wild_bee	1	(0.04)	1
I_14	*Andrena fulva*	Wild_bee	2	(0.07)	4
I_15	*Andrena gravida*	Wild_bee	8	(0.29)	1
I_16	*Andrena haemorrhoa*	Wild_bee	44	(1.58)	1
I_17	*Andrena helvola*	Wild_bee	8	(0.29)	5
I_18	*Andrena labiata*	Wild_bee	1	(0.04)	8
I_19	*Andrena minutula*	Wild_bee	3	(0.11)	13
I_20	*Andrena minutuloides*	Wild_bee	4	(0.14)	11
I_21	*Andrena nigroaenea*	Wild_bee	29	(1.04)	5
I_22	*Andrena nitida*	Wild_bee	6	(0.22)	1
I_23	*Andrena scotica*	Wild_bee	2	(0.07)	3
I_24	*Andrena strohmella*	Wild_bee	6	(0.22)	1
I_25	*Andrena subopaca*	Wild_bee	2	(0.07)	8
I_26	*Andrena synadelpha*	Wild_bee	1	(0.04)	5
I_27	*Andrena vaga*	Wild_bee	1	(0.04)	1
I_28	*Andrena varians*	Wild_bee	1	(0.04)	3
I_29	*Andrena viridescens*	Wild_bee	3	(0.11)	8
I_30	*Anthaxia nitidula*	Coleoptera	12	(0.43)	16
I_31	*Anthocharis cardamines*	Lepidoptera	2	(0.07)	9
I_32	Anthomyiidae	Other_fly	60	(2.16)	5
I_33	*Anthophora plumipes*	Wild_bee	41	(1.48)	4

I_34	*Apis mellifera*	Honeybee	361	(12.99)	5
I_35	*Araschnia levana*	Lepidoptera	1	(0.04)	2
I_36	Asilidae	Other_fly	2	(0.07)	10
I_37	*Athalia rosae*	Wasp	1	(0.04)	10
I_38	Bibionidae	Other_fly	40	(1.44)	3
I_39	*Bombus dark form complex*	Bumblebee	9	(0.32)	5
I_40	*Bombus hortorum*	Bumblebee	1	(0.04)	4
I_41	*Bombus hypnorum*	Bumblebee	1	(0.04)	5
I_42	*Bombus lapidarius*	Bumblebee	1	(0.04)	5
I_43	*Bombus pascuorum*	Bumblebee	235	(8.46)	4
I_44	*Bombus pratorum*	Bumblebee	5	(0.18)	4
I_45	*Bombus red-tail complex*	Bumblebee	46	(1.66)	5
I_46	*Bombus terrestris*	Bumblebee	4	(0.14)	4
I_47	*Bombus terrestris complex*	Bumblebee	238	(8.57)	4
I_48	*Bombylius major*	Other_fly	121	(4.36)	8
I_49	*Brachyderini sp 1*	Coleoptera	2	(0.07)	16
I_50	*Brachyderini sp 2*	Coleoptera	1	(0.04)	1
I_51	*Byturus ochraceus*	Coleoptera	35	(1.26)	2
I_52	Calliphoridae	Other_fly	17	(0.61)	7
I_53	*Cantharis fusca*	Coleoptera	13	(0.47)	15
I_54	*Cephus brachycerus*	Wasp	32	(1.15)	2
I_55	*Cephus pygmeus*	Wasp	56	(2.02)	9
I_56	*Ceratina cyanea*	Wild_bee	2	(0.07)	2
I_57	*Cetonia aurata*	Coleoptera	32	(1.15)	5
I_58	*Cheilosia illustrata*	Hoverfly	1	(0.04)	10
I_59	*Cheilosia latifrons*	Hoverfly	12	(0.43)	2
I_60	*Cheilosia vicina*	Hoverfly	2	(0.07)	2
I_61	*Chelostoma florisomne*	Wild_bee	1	(0.04)	2
I_62	*Chrysanthia sp*	Coleoptera	1	(0.04)	1
I_63	*Chrysomela aenea*	Coleoptera	2	(0.07)	4
I_64	*Chrysotoxum cautum*	Hoverfly	9	(0.32)	10
I_65	*Chrysotoxum festivum*	Hoverfly	2	(0.07)	15
I_66	*Cladius pectinicornis*	Wasp	1	(0.04)	5
I_67	*Coccinella septempunctata*	Coleoptera	6	(0.22)	5
I_68	*Coenonympha pamphilus*	Lepidoptera	11	(0.4)	10
I_69	*Colias alfacariensis*	Lepidoptera	1	(0.04)	1
I_70	*Colletes cunicularius*	Wild_bee	6	(0.22)	3
I_71	Collyriinae	Wasp	1	(0.04)	10
I_72	Conopidae	Other_fly	5	(0.18)	3
I_73	*Dasysyrphus albostriatus*	Hoverfly	2	(0.07)	1
I_74	*Dasysyrphus tricinctus*	Hoverfly	4	(0.14)	1

I_75	*Dasysyrphus venustrus*	Hoverfly	2	(0.07)	3
I_76	*Dasytes virens*	Coleoptera	1	(0.04)	5
I_77	*Dinoptera collaris*	Coleoptera	2	(0.07)	10
I_78	Dixidae	Other_fly	7	(0.25)	1
I_79	Elateridae	Coleoptera	33	(1.19)	15
I_80	*Elinora koehleri*	Wasp	3	(0.11)	13
I_81	*Ematurga atomaria*	Lepidoptera	1	(0.04)	1
I_82	Empididae	Other_fly	171	(6.16)	1
I_83	Entiminae	Coleoptera	3	(0.11)	1
I_84	*Epistrophe eligans*	Hoverfly	1	(0.04)	5
I_85	*Episyrphus balteatus*	Hoverfly	1	(0.04)	5
I_86	*Eristalis arbustorum*	Hoverfly	1	(0.04)	13
I_87	*Eristalis horticola*	Hoverfly	1	(0.04)	14
I_88	*Eristalis jugorum*	Hoverfly	1	(0.04)	1
I_89	*Eristalis similis*	Hoverfly	1	(0.04)	10
I_90	*Eristalis tenax*	Hoverfly	6	(0.22)	5
I_91	*Eucera nigrescens*	Wild_bee	1	(0.04)	6
I_92	*Eupeodes corollae*	Hoverfly	1	(0.04)	10
I_93	*Eupeodes luniger*	Hoverfly	1	(0.04)	5
I_94	*Gnorimus variabilis*	Coleoptera	1	(0.04)	5
I_95	*Gonepteryx rhamni*	Lepidoptera	3	(0.11)	9
I_96	*Halictus maculatus*	Wild_bee	3	(0.11)	7
I_97	*Halictus scabiosae*	Wild_bee	2	(0.07)	10
I_98	*Halictus tumulorum*	Wild_bee	10	(0.36)	5
I_99	*Helophilus pendulus*	Hoverfly	2	(0.07)	1
I_100	Ichneumoninae	Wasp	2	(0.07)	1
I_101	*Issoria lathonia*	Lepidoptera	1	(0.04)	1
I_102	*Lasioglossum albipes*	Wild_bee	1	(0.04)	1
I_103	*Lasioglossum calceatum*	Wild_bee	18	(0.65)	1
I_104	*Lasioglossum fulvicorne*	Wild_bee	1	(0.04)	16
I_105	*Lasioglossum laevigatum*	Wild_bee	1	(0.04)	9
I_106	*Lasioglossum lativentre*	Wild_bee	1	(0.04)	1
I_107	*Lasioglossum lineare*	Wild_bee	1	(0.04)	8
I_108	*Lasioglossum malachurum*	Wild_bee	7	(0.25)	1
I_109	*Lasioglossum nitidiusculum*	Wild_bee	2	(0.07)	4
I_110	*Lasioglossum pallens*	Wild_bee	1	(0.04)	5
I_111	*Lasioglossum parvulum*	Wild_bee	3	(0.11)	13
I_112	*Lasioglossum pauxillum*	Wild_bee	20	(0.72)	9
I_113	*Lasioglossum politum*	Wild_bee	1	(0.04)	5
I_114	*Lasioglossum rufitarse*	Wild_bee	1	(0.04)	1
I_115	*Lasioglossum sp*	Wild_bee	4	(0.14)	5

I_116	*Lasioglossum villosulum*	Wild_bee	1	(0.04)	1
I_117	*Leptidea sinapis*	Lepidoptera	1	(0.04)	5
I_118	*Longitarsus sp*	Coleoptera	1	(0.04)	1
I_119	*Macrophya montana*	Wasp	1	(0.04)	7
I_120	*Malachius bipustulatus*	Coleoptera	10	(0.36)	5
I_121	*Melanogaster hirtella*	Hoverfly	1	(0.04)	1
I_122	*Melanostoma mellinum*	Hoverfly	6	(0.22)	5
I_123	*Melanostoma scalare*	Hoverfly	3	(0.11)	1
I_124	Mordellidae	Coleoptera	170	(6.12)	5
I_125	Muscidae	Other_fly	11	(0.4)	9
I_126	*Nomada bifasciata*	Wild_bee	14	(0.5)	1
I_127	*Nomada fabriciana*	Wild_bee	1	(0.04)	1
I_128	*Nomada flava*	Wild_bee	5	(0.18)	3
I_129	*Nomada flavoguttata*	Wild_bee	2	(0.07)	1
I_130	*Nomada fucata*	Wild_bee	2	(0.07)	10
I_131	*Nomada goodeniana*	Wild_bee	33	(1.19)	1
I_132	*Nomada lathburiana*	Wild_bee	4	(0.14)	1
I_133	*Nomada marshamella*	Wild_bee	7	(0.25)	4
I_134	*Nomada panzeri*	Wild_bee	12	(0.43)	3
I_135	*Nomada ruficornis*	Wild_bee	4	(0.14)	1
I_136	*Nomada sp*	Wild_bee	1	(0.04)	8
I_137	*Nomada succincta*	Wild_bee	5	(0.18)	3
I_138	*Oedemera lurida*	Coleoptera	3	(0.11)	1
I_139	*Oedemera virescens*	Coleoptera	8	(0.29)	2
I_140	*Osmia bicolor*	Wild_bee	1	(0.04)	5
I_141	*Osmia bicornis*	Wild_bee	3	(0.11)	5
I_142	*Osmia brevicornis*	Wild_bee	25	(0.9)	9
I_143	*Pachyprotasis rapae*	Wasp	1	(0.04)	14
I_144	*Parasyrphus annulatus*	Hoverfly	1	(0.04)	14
I_145	*Phloeopora sp*	Coleoptera	1	(0.04)	1
I_146	*Phyllobius sp*	Coleoptera	4	(0.14)	5
I_147	*Pieris brassicae*	Lepidoptera	5	(0.18)	7
I_148	*Pieris napi*	Lepidoptera	7	(0.25)	9
I_149	*Pipizella divicoi*	Hoverfly	1	(0.04)	16
I_150	*Pipizella viduata*	Hoverfly	2	(0.07)	16
I_151	*Pipizella zeneggenesis*	Hoverfly	1	(0.04)	10
I_152	*Pissodes sp*	Coleoptera	3	(0.11)	3
I_153	*Platycheirus albimanus*	Hoverfly	18	(0.65)	8
I_154	*Platycheirus scutatus*	Hoverfly	3	(0.11)	7
I_155	*Platycheirus sp*	Hoverfly	1	(0.04)	3
I_156	*Platycheirus tarsalis*	Hoverfly	2	(0.07)	4

I_157	*Podistra sp*	Coleoptera	1	(0.04)	2
I_158	*Poecilus cupreus*	Coleoptera	1	(0.04)	1
I_159	*Poecilus versicolor*	Coleoptera	2	(0.07)	5
I_160	*Polyommatus bellargus*	Lepidoptera	3	(0.11)	4
I_161	*Pontia edusa*	Lepidoptera	3	(0.11)	4
I_162	*Procraerus tiblalis*	Coleoptera	1	(0.04)	10
I_163	Pyralidae	Lepidoptera	9	(0.32)	14
I_164	*Pyrgus malvae*	Lepidoptera	2	(0.07)	9
I_165	Rhagionidae	Other_fly	1	(0.04)	5
I_166	Sarcophagidae	Other_fly	38	(1.37)	5
I_167	*Scaeva selenitica*	Hoverfly	5	(0.18)	5
I_168	*Sphaerophoria rueppellii*	Hoverfly	1	(0.04)	1
I_169	*Sphaerophoria scripta*	Hoverfly	7	(0.25)	13
I_170	*Sphecodes albilabris*	Wild_bee	2	(0.07)	3
I_171	*Sphecodes ephippius*	Wild_bee	2	(0.07)	1
I_172	*Sphecodes spinulosus*	Wild_bee	1	(0.04)	15
I_173	*Syrphus ribesii*	Hoverfly	21	(0.76)	8
I_174	*Syrphus torvus*	Hoverfly	35	(1.26)	5
I_175	*Syrphus vitripennis*	Hoverfly	6	(0.22)	5
I_176	*Tachina fera*	Other_fly	1	(0.04)	1
I_177	Tachinidae	Other_fly	19	(0.68)	2
I_178	*Tenthredo sulphuripes*	Wasp	1	(0.04)	5
I_179	*Tenthredo temula*	Wasp	1	(0.04)	5
I_180	*Tenthredo zonula*	Wasp	5	(0.18)	7
I_181	*Tenthredopsis sp*	Wasp	5	(0.18)	10
I_182	Tipulidae	Other_fly	3	(0.11)	14
I_183	*Tropinota hirta*	Coleoptera	12	(0.43)	1
I_184	Tryphoninae	Wasp	8	(0.29)	7
I_185	Ulidiidae	Other_fly	2	(0.07)	10
I_186	*Valgus hemipterus*	Coleoptera	2	(0.07)	7
I_187	*Vesperus sp*	Coleoptera	1	(0.04)	3
I_188	*Vespula rufa*	Wasp	1	(0.04)	1
I_189	*Xantogramma pedissequum*	Hoverfly	1	(0.04)	13
